# Vanadium Toxicity Monitored by Fertilization Outcomes and Metal Related Proteolytic Activities in *Paracentrotus lividus* Embryos

**DOI:** 10.3390/toxics10020083

**Published:** 2022-02-10

**Authors:** Roberto Chiarelli, Chiara Martino, Maria Carmela Roccheri, Fabiana Geraci

**Affiliations:** Department of Biological, Chemical and Pharmaceutical Sciences and Technologies (STEBICEF), University of Palermo, Viale delle Scienze Building 16, 90128 Palermo, Italy; chiaracomlib@yahoo.it (C.M.); maria.roccheri@unipa.it (M.C.R.)

**Keywords:** sodium orthovanadate, sea urchin embryos, gelatinases, metalloproteinases

## Abstract

Metal pharmaceutical residues often represent emerging toxic pollutants of the aquatic environment, as wastewater treatment plants do not sufficiently remove these compounds. Recently, vanadium (V) derivatives have been considered as potential therapeutic factors in several diseases, however, only limited information is available about their impact on aquatic environments. This study used sea urchin embryos (*Paracentrotus lividus*) to test V toxicity, as it is known they are sensitive to V doses from environmentally relevant to very cytotoxic levels (50 nM; 100 nM; 500 nM; 1 µM; 50 µM; 100 µM; 500 µM; and 1 mM). We used two approaches: The fertilization test (FT) and a protease detection assay after 36 h of exposure. V affected the fertilization percentage and increased morphological abnormalities of both egg and fertilization envelope, in a dose-dependent manner. Moreover, a total of nine gelatinases (with apparent molecular masses ranging from 309 to 22 kDa) were detected, and their proteolytic activity depended on the V concentration. Biochemical characterization shows that some of them could be aspartate proteases, whereas substrate specificity and the Ca^2+^/Zn^2+^ requirement suggest that others are similar to mammalian matrix metalloproteinases (MMPs).

## 1. Introduction

Metal toxicology is a topic of considerable importance for two main reasons. First, several metals are widely used both in industrial and therapeutic fields. Second, once released into the environment, metals bioaccumulate in living organisms because of the lack of specific excretion mechanisms [1]. Metal toxicity was reported from aquatic to terrestrial organisms, such as sea urchins, polychaetes, and birds [1,2,3]. Although metal industrial use has been partially reduced due to the introduction of alternative materials, their interest in the clinical field is rapidly growing, opening a new topic of research called metalpharmaceuticals (metal-containing pharmaceutics). Many metals have already a notable clinical interest due to their application as confirmed metallodrugs, such as platinum, copper, gold, lithium, ruthenium, and yttrium. Others are instead used in medical diagnosis, for example gadolinium [4,5].

Some metallodrugs are anticipated to be developed as pharmaceuticals, among them germanium, molybdenum, selenium, and vanadium (V) [6].

V is a trace element extensively distributed in nature, and present in almost all living organisms, including man [7]. V derivatives are used in various heavy industries (e.g., steel and oil). Around 85% of the produced global V is used as ferrovanadium. The incidence of industrial worker exposure to V toxic levels has been increasing, highlighting several harmful effects [8]. Among the toxicologically significant V compounds there are vanadium pentoxide (V_2_O_5_), sodium metavanadate (Na_3_VO_3_), sodium orthovanadate (Na_3_VO_4_), vanadyl sulfate (VOSO_4_), and ammonium vanadate (NH_4_VO_3_) [9]. These compounds are well known environmental pollutants and their toxicity involves oxidative stress [10]. As wastewater treatment plants are not designed to sufficiently remove these compounds [11], they are also becoming emerging toxic pollutants of aquatic environments.

Recently, V derivatives have been considered as potential therapeutic factors for several diseases (e.g., obesity, diabetes, cancer, neurodegenerative, and heart disorders) [12,13,14]. For all these reasons, many studies on V compounds were conducted to investigate their therapeutic and/or toxicological effects [15]. There is a considerable interest on Na_3_VO_4_ due to its role in the treatment of diabetes [12], even if its toxic effects in terms of stress, autophagy, and apoptosis induction were recently reported [16]. The V_2_O_5_ is instead used as a catalyst for the conversion of sulphur dioxide to trioxide when producing sulphuric acid and maleic anhydride, which is a chemical required to produce polyester resins and fiberglass [17]. It is probably carcinogenic to humans since it can generate ROS, and cause oxidative DNA damage, which may lead to mutations and cancer promotion [18]. Dangerous effects on cytochrome p450 activities, DNA damage, and DNA methylation were recently reported for Na_3_VO_3_ in human liver cell lines [19]. Genotoxic and cytotoxic effects of VOSO_4_ were observed in rats where this compound induces hepatocellular toxicity, oxidative stress, and damage to the DNA [20].

All these studies suggest that although there are promising therapeutic effects for V compounds, further studies on their noxious effects are still necessary to clarify the toxicological profiles [16].

Knowledge about V environmental effects is still relatively scarce [21]. Considering the relevance of this problem, researches on its toxic effects need great attention. For this reason, to study V toxicity we used a well-known model system, the sea urchin *Paracentrotus lividus* (*P. lividus*) embryos. This model has already been successfully used to determine the cyto-toxicity of several metals (e.g., Cd, Gd, Ni, Pb, Cu, and Zn) [1,16,22,23,24,25,26,27,28,29,30,31,32,33,34], and has been recently confirmed as an adequate system for detecting V noxious effects [16].

It was already demonstrated that V induces in sea urchin embryos a precise toxicological response, modulated by heat shock proteins, autophagy, and apoptosis. These are specific pathways involved both in embryo physiological development and stress [35,36,37]. Furthermore, skeleton damages were observed, suggesting possible V competition with calcium [16].

It is also known that V, both as an anticancer drug and as a toxic pollutant, is able to regulate in different organisms, such as human and mice the expression of matrix metalloproteinases (MMPs), especially MMP2 and MMP9, a family of Zn^2+^ and Ca^2+^ -dependent proteinases [38,39,40,41].

Extracellular matrix remodeling is fundamental for correct embryogenesis, in particular for cell migration. During early gastrulation of sea urchin embryos, primary mesenchyme cells ingress into the blastocoel, where they undergo fusion to form a syncytium involved in skeleton formation [35,42]. Roe and co-workers have demonstrated that metalloproteinases inhibition blocked spicule formation both in vivo and in vitro [43]. These data were later confirmed by Ingersoll et al. [44,45].

Chemical toxicology, especially related to metals, requires extensive investigation to define their toxicological profiles. The final aim of this paper is to increase the knowledge about the toxicological profile of V in sea urchin embryos, with reference to biological processes directly dependent on the action of metals. Here we demonstrate that both sea urchin fertilization and metalloproteinase activity are markedly perturbed, indicating that they can represent specific markers of V toxicity. In particular, V affects fertilization envelope morphology and modulates levels and typologies of gelatinases, in a dose dependent manner.

## 2. Materials and Methods

### 2.1. Gamete Collection

Adult specimens of *P. lividus* were collected in shallow rocky reefs (2–10 m) along the coast of the marine protected area (MPA) of Favignana island (Trapani, Sicily, Italy). They were immediately brought to the laboratory and used to isolate eggs and sperms from gonads. The experiments were repeated three times using gametes pooled from multiple males and females.

Eggs from each female were quickly collected, suspended in natural Millipore filtered sea water (MFSW), and inspected under an optical microscope. Samples containing vacuolated, irregular, and small eggs were discarded and only those containing perfectly spherical eggs were selected. Aliquots of these eggs were subjected to a rapid pre-fertilization test by adding to each representative sub-sample (100 eggs) of each group emitted by each individual specimen, a small quantity of sperm diluted in MFSW (1 × 10^6^ sperms). Groups of eggs not fully fertilized in a short time (1 min) were discarded. Selected eggs were then pooled in a glass beaker. During the whole operation, the temperature was 18 ± 1 °C.

The sperm was recovered without sea water (dry), directly from the animal’s gonads, and stored at 4 °C. Before the fertilization test, sperm maturity (mobile spermatozoa) was checked by observation under a microscope.

### 2.2. Sodium Orthovanadate Stock Solution Preparation

Sodium orthovanadate (Na_3_VO_4_, Sigma-Aldrich, St. Louis, MO, USA) stock solution (0.1 M) was prepared according to the manufacturer’s instructions. Briefly, it was prepared in distilled water adjusted to pH 10. To ensure the presence of vanadate monomers, the solution was boiled until translucent and the pH was readjusted to 10. Before boiling, the solution appeared yellow/orange due to the decavanadate presence. The absence of decavanadate in the solution was confirmed by reading the stock solution (1:100 diluted) from 220 to 700 nm, by a Cary 100 UV-Visible Spectrophotometer.

The different V concentrations were obtained by dilutions from this stock solution.

### 2.3. Fertilization Test

The fertilization test was conducted according to Carbailleira and co-workers [46], with some modifications. Briefly, to begin, a concentration of 5000 eggs/mL was diluted (1:250) and 20 mL of this suspension (400 eggs) was placed in plastic Petri dishes, 90 mm in diameter, and used for each experimental point. Aliquots of egg suspension were maintained as the control, while other aliquots were treated for 60 min with eight different V concentrations (50 nM; 100 nM; 500 nM; 1 µM; 50 µM; 100 µM; 500 µM; and 1 mM).

A volume of 200 µL of a suspension of 4 × 10^6^ sperm was added to each egg sample. Fertilization was allowed to take place for 25 min and subsequently blocked adding a drop of 40% formaldehyde in MFSW. Results of microscope observation were reported after classifying 100 eggs according to these categories: (a) eggs with normal morphology and normal fertilization membrane; (b) eggs with normal morphology and abnormal fertilization membrane; (c) eggs with abnormal morphology and normal fertilization membrane; (d) eggs with abnormal morphology and abnormal fertilization membrane; (e) unfertilized eggs with normal morphology; and (f) unfertilized eggs with abnormal morphology.

Based on the above categories, we then obtained a Toxicity Index (TI) according to Ramdial et al. [47]. Briefly, we assigned the following scores according to the degree of anomaly: 0 for eggs with normal morphology and normal fertilization membrane, 1 for eggs with normal morphology and abnormal fertilization membrane plus eggs with abnormal morphology and normal fertilization membrane, 2 for eggs with abnormal morphology and abnormal fertilization membrane, and 3 for unfertilized eggs with normal morphology plus unfertilized eggs with abnormal morphology.

The following equation was used to define TI:

TI = [(0 × %Type 0) + (1 × %Type 1) + (2 × %Type 2) + (3 x× %Type 3)]/100.

Morphological analyses were carried out under 20× objectives of an Olympus Bx50 microscope and photographed with a digital camera (Nikon Sight DS-U1, Nikon, Tokyo, Japan).

### 2.4. Embryo Cultures and V Treatments

Embryo cultures and V treatments were carried out as described by Chiarelli et al. [16]. Briefly, after fertilization, embryos were maintained at 18 °C in glass containers with gentle mixing. We used 5 × 10^4^ embryos for each experimental condition. The embryonic development time, corresponding to the exposure time, was 36 h.

### 2.5. Gelatin Zymography by Polyacrylamide Gel Electrophoresis

Pellets of control and V-treated embryos, obtained by embryo culture centrifugation at 800 g, were lysed with buffer containing 20 mM Tris, pH 7.4; 150 mM NaCl; and 0.5% Triton X-100. No proteases inhibitors were added.

The lysis was undertaken by subjecting embryonic pellets to 3 cyclic treatments consisting of 30 s in liquid nitrogen and 4 min at 37 °C. Afterwards, the samples were centrifuged (35,000× *g* at 4 °C) and the supernatants were stored at −80 °C until used.

A total of 15 µg of total cell lysates, determined by the Bradford method, were analyzed by 10% SDS-PAGE gel zymography as described by Pinsino et al. [48].

Zymogramme band densities and the molecular weight of each gelatinase were determined using the Quantity One 4.6.5 software (Bio-Rad, Hercules, CA, USA).

The biochemical characterization of each gelatinase was obtained by using the following inhibitors: 2 mM EDTA; 10 mM EGTA; 2 mM 1,10 phenanthroline; 10 mM DTT; 2 mM PMSF; and 1 µM pepstatine A in gels loaded with controls and V treated (100 nM) lysates.

### 2.6. Statistical Analysis

Data for each of the morphological categories obtained from three independent fertilization tests were analyzed by one-way analysis of variance (ANOVA). Levene’s test was used to check the homogeneity of variance. Tukey’s honestly significant difference (Tukey’s HSD) test was employed as a post-hoc test for mean comparison to evaluate significative differences between data derived from controls and V-treated eggs. The analyses were performed using the Statistica 13.2 software (StatSoft, Tulsa, OK, USA), and the level of statistical significances was set to *p* < 0.05 (*), *p* < 0.01 (**) and *p* < 0.001 (***).

Values of relative gelatinase activities of embryos obtained from three independent fertilizations were analyzed by unpaired two-tailed Student’s t-test. The analyses were performed with GraphPad Prism 9 software (GraphPad). The statistical significances were set to *p* ≤ 0.05 (*), *p* ≤ 0.01 (**), and *p* ≤ 0.0005 (***).

All data are represented as means of three independent experiments (*n* = 3) ± standard deviation (SD).

## 3. Results

### 3.1. V Reduces the Fertilization Rate and Increases Fertilized Egg Anomalies

**Figure 1:**
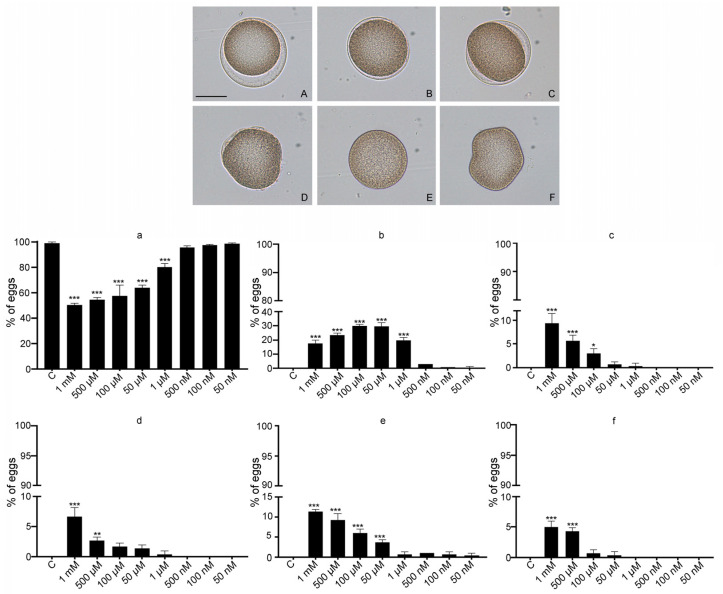
Effects of V exposure on the percentage of fertilization events in the sea urchin *Paracentrotus lividus*. Upper panel: images of representative eggs captured by light microscopy. Egg with normal morphology and normal fertilization membrane (**A**). Egg with normal morphology and abnormal fertilization membrane (**B**). Egg with abnormal morphology and normal fertilization membrane (**C**). Egg with abnormal morphology and abnormal fertilization membrane (**D**). Unfertilized egg with normal morphology (**E**). Unfertilized egg with abnormal morphology (**F**). Bar = 50 µm. Lower panel: histogram bars showing the percentage of the number of eggs with each morphology per total of eggs used in each treatment. % of eggs with normal morphology and normal fertilization membrane (**a**). % of eggs with normal morphology and abnormal fertilization membrane (**b**). % of eggs with abnormal morphology and normal fertilization membrane (**c**). % of eggs with abnormal morphology and abnormal fertilization membrane (**d**). % of unfertilized eggs with normal morphology (**e**). % of unfertilized eggs with abnormal morphology (**f**). The statistical significances was set to *p* < 0.05 (*), *p* < 0.01 (**) and *p* < 0.001 (***).

As fertilization and early developmental stages in the life of marine animals are highly sensitive to environmental perturbations, we firstly determined the fertilization rate after V exposure. Treatment of virgin eggs with different V concentrations reduced fertilization success (F_(8,18)_: 41.8, *p* = 0.006), measured by the presence or absence of the fertilization membrane. Control eggs showed a fertilization rate of 99.5 ± 0.5%, while for V-treated eggs the fertilization success was: 1 mM, 84 ± 1.8%; 500 µM, 87 ± 1.5%; 100 µM, 93 ± 0.5%; 50 µM, 96 ± 0.5%; 1 µM, 99 ± 0.5%; 500 nM, 99 ± 0.3%; 100 nM, 99 ± 0.3%; and 50 nM, 99 ± 0.5%, showing that the reduction depended on the V concentration. Although most of the V-treated eggs showed a fertilization membrane, its presence did not seem to be a reliable index reflecting V damage since we also observed abnormalities in the morphology of the egg and/or of the fertilization membrane. For this reason, an additional method of quantifying V toxicity was applied. After fertilization, control eggs showed a normal morphology and a correct fertilization membrane phenotype (i.e., perfectly spherical eggs with a fertilization membrane totally detached from its surface) (Figure 1A), and also parts of V-treated eggs showed this morphology. In contrast, most of the V-treated eggs displayed a significant reduction in these parameters (F_8,18_: 4132.03; *p* = 0.002) compared to controls, (Tukey’s HSD: C > 1 mM, 500 µM, 100 µM, 50 µM, and 1 µM) (Figure 1A, histogram a). V-exposed eggs showed the following alterations: Egg with normal morphology and abnormal fertilization membrane (F_(8,18)_: 234.6; *p* = 0.009, Tukey’s HSD: C < 1 mM, 500 µM, 100 µM, 50 µM, and 1 µM) (Figure 1B, histogram b); egg with abnormal morphology and normal fertilization membrane (F_(8,18)_: 40.8; *p* = 0.0007, Tukey’s HSD: C < 1 mM, 500 µM, and 100 µM) (Figure 1C, histogram c); egg with abnormal morphology and abnormal fertilization membrane, (F_(8,18)_: 35.34; *p* = 0.0008, Tukey’s HSD: C < 1 mM, 500 µM) (Figure 1D, histogram d); unfertilized egg with normal morphology, (F_(8,18)_: 98.55; *p* = 0.02, Tukey’s HSD: C < 1 mM, 500 µM, 100 µM, and 50 µM) (Figure 1E, histogram e); and unfertilized egg with abnormal morphology, (F_(8,18)_: 54.79 *p* = 0.003, Tukey’s HSD: C < 1 mM, 500 µM) (Figure 1F, histogram f).

Our data demonstrate that the fertilization index (fertilized vs. unfertilized) is not a reliable parameter to monitor V toxicity by itself. However, our approach, reporting a more precise observation of the obtained phenotypes, clearly demonstrates that V affects different aspects of fertilization and it could represent a rapid and suitable marker of metal toxicity.

Results of TI, related to the above categories are reported in Table 1.

### 3.2. V Perturbs the Proteolytic Activities of Gelatinases

As previously described by Chiarelli et al., V can influence sea urchin embryos not only by delaying their development, but also by altering skeleton formation [16]. Furthermore, it is well known that V influences MMP activity [38,39,40,41], whose expression is also involved in sea urchin embryo skeletogenesis [43,44,45]. For these reasons, we tested the gelatinolytic activity of control and V-treated embryos. Using gelatine substrate gel zymography, a dynamic pattern of cleavage activities was identified.

Control embryos showed the proteolytic activity of 9 distinct gelatinases (named according to their apparent molecular masses 309, 255, 177, 79, 59, 34, 30, 25, and 22 kDa). After 36 h of exposure from fertilization, embryos treated with different V concentrations showed both a different pattern of expression and a different level of activity of gelatinases.

**Figure 2:**
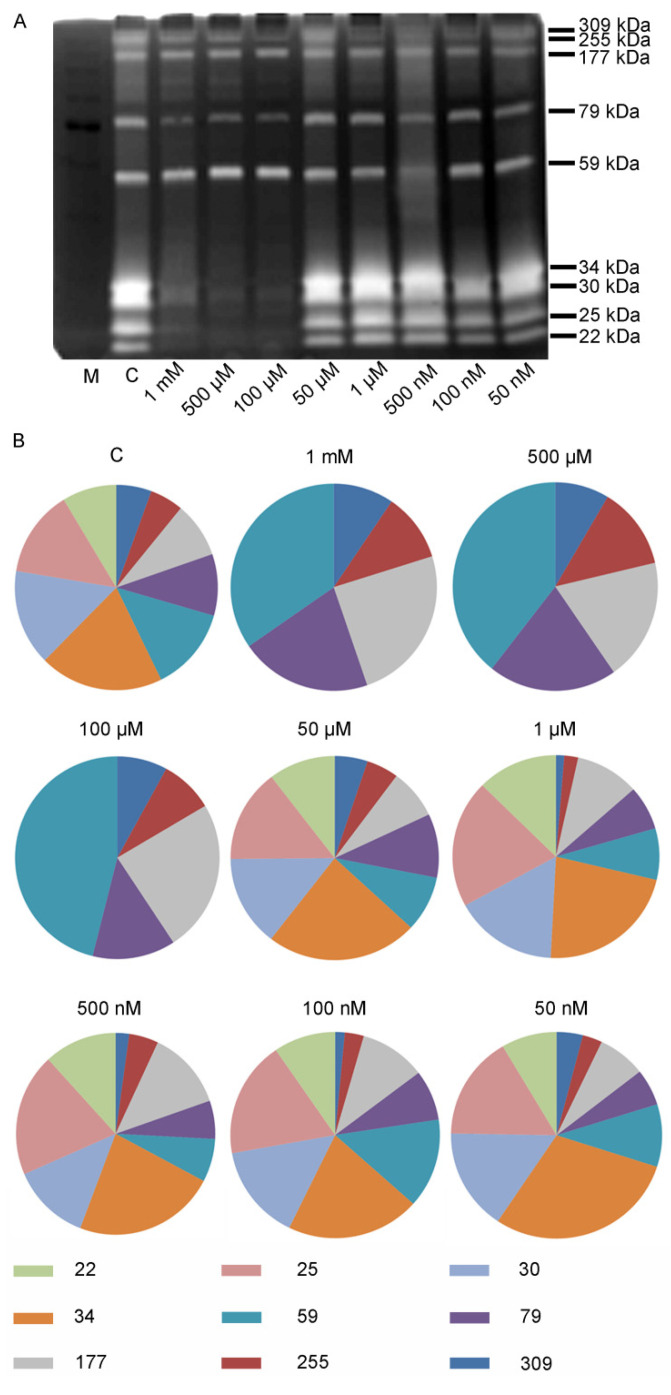
Proteolytic activities analyzed by gelatin substrate gel zimography. (**A**) Zimogram showing gelatinase bands in lysates of embryos at 36 h of growth from: control and V-treated embryos. M = protein molecular weight marker. (**B**) The pie charts display the percentages of each gelatinase activity (309, 225, 177, 79, 59, 34, 30, 25, and 22 kDa), for control and treated embryos after 36 h of development.

Embryos exposed to very high V concentrations (100 µM, 500 µM, and 1 mM) did not show any proteolytic activity mediated by the gelatinases 34, 30, 25, and 22 kDa. On the other hand, these embryos showed an increased activity for gelatinases 309, 255, 177, 79, and 59 kDa. In contrast, embryos exposed to lower V concentrations (50 µM, 1 µM, 500 nM, 100 nM, and 50 nM) showed a proteolytic activity comparable to that of control embryos for both typology and quantity (Figure 2A,B).

### 3.3. Relative Gelatinase Activities

**Figure 3:**
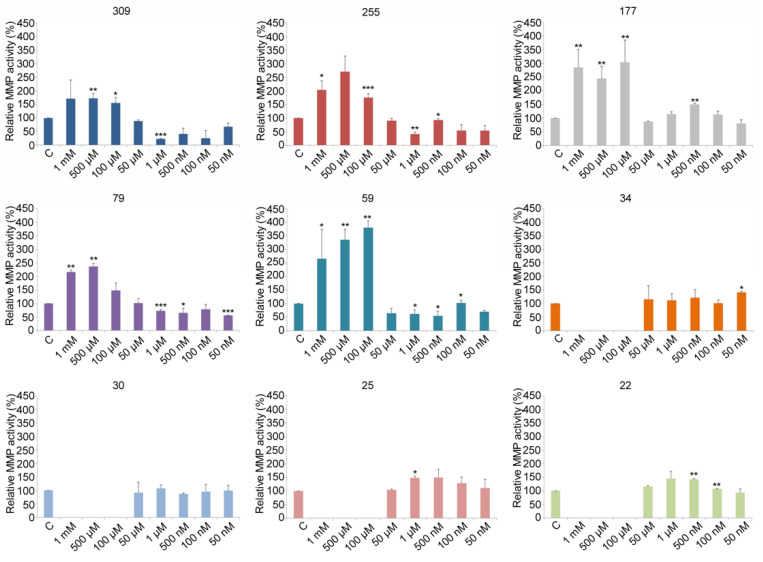
Relative gelatinase activities. The histograms show the percentage of relative gelatinase activity for each gelatinase in control and V-treated embryos after 36 h of development. All values were normalized with respect to the gelatinases activity of the control samples (fixed to 100%). The statistical significances were set to *p* ≤ 0.05 (*), *p* ≤ 0.01 (**), and *p* ≤ 0.0005 (***).

The preceding results demonstrated that V induces dose dependent perturbations in each analyzed gelatinase activity. In particular, high V concentrations (100 µM, 500 µM, and 1 mM) induced an increased level of activity for high molecular weight gelatinases (i.e., 309, 255, 177, 79, and 59 kDa), and completely inhibited the proteolytic activities of the low molecular weight proteases (i.e., 34, 30, 25, and 22 kDa) (Figure 3).

Zymography assays showed that the 309 kDa protease activity increased compared to controls in embryos exposed to the highest V concentrations (100 µM, 500 µM, and 1 mM), without marked differences among the three concentrations. In contrast, protease activity drastically decreased in embryos exposed to the lowest V concentrations (50 nM, 100 nM, 500 nM, 1 µM, and 50 µM) (Figure 3).

Similarly, the relative 255 kDa protease activity increased in embryos exposed to the highest V concentrations, especially at 500 µM V. Conversely, its activity level decreased in embryos exposed to the lowest V concentrations (Figure 3).

Different from the two previous gelatinases, the relative 177 kDa activity significantly increased with respect to controls in embryos exposed to all the three highest V concentrations, whereas in the lowest V ones, levels remained similar to control embryos.

The 79 and 59 kDa gelatinases were similarly influenced by the V treatment. In particular, their gelatinolytic activities, especially that of 59 kDa, were significantly increased by high V concentrations. By contrast, a statistically significant reduction was observed for the embryos treated with the lowest V concentrations (Figure 3).

A completely different behavior was observed for the lowest gelatinases (i.e., 34, 30, 25, and 22 kDa). High V concentrations induced a complete inhibition of their proteolytic activity. No signal of these enzymatic activities was observed for embryos exposed to 100 µM, 500 µM, and 1 mM, respectively. In contrast, no substantial differences were observed in embryos exposed to the lowest V concentrations (Figure 3).

### 3.4. Gelatinase Characterization Using Chemical Inhibitors

**Figure 4:**
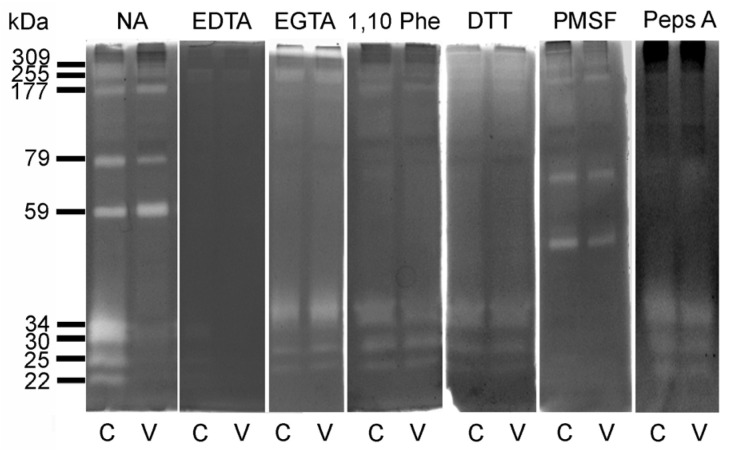
Proteolytic activities analyzed by gelatin substrate gel zimography for control (C) and V-treated (V) embryos. The analysis was conducted in the absence (NA) or in the presence of a variety of protease inhibitors: Ethylenediaminetetraacetic acid (EDTA); ethylene glycol-bis (β-aminoethyl ether)-N,N,N′,N′-tetraacetic acid (EGTA); 1,10 phenanthroline (1,10-Phe); Dithiothreitol (DTT); Phenylmethylsulfonyl fluoride (PMSF); pepstatin A (peps A).

In order to biochemically characterize the gelatinases observed after V exposure, a series of chemical inhibitors were employed, and the effects on their activities were determined. Lysates of both control and V-treated embryos (100 nM) were loaded onto SDS gelatin substrate gel to test their proteolytic activity in the presence or absence of EDTA, a nonspecific divalent metal ion chelator (i.e., Ca^2+^, Mg^2+^, and Zn^2+^). When it was added in the incubation buffer, a drastic reduction in the activity of all the gelatinases, especially the 79 and 59 kDa, was observed (Figure 4).

To better characterize divalent ion involvement, gelatin zymography was undertaken by incubating gels with either EGTA, a Ca^2+^ chelator, or 1,10-phenanthroline, a Zn^2+^-specific chelator. When EGTA was added to the incubation buffer, the gelatin degrading activities corresponding to 79 and 59 kDa proteases ceased, and a severe reduction in 177 kDa was observed. Furthermore, the addition of 1,10-phenanthroline caused the complete inhibition of 79 and 59 kDa gelatinases (Figure 4).

The identified proteases were further characterized by determining the effects of different protease inhibitors. DTT, which reduces disulfide bonds, blocks the gelatin degrading activities of both 79 and 59 kDa. These results collectively suggest that all the observed gelatinases depend on divalent ions, and in particular the 79 and 59 kDa proteases require both Ca^2+^ and Zn^2+^, as well as an intact tertiary structure, for their activity (Figure 4).

The irreversible protease inhibitor, PMSF, causes a complete disappearance of the lowest molecular weight gelatinases (i.e., 34, 30, 25, and 22 kDa), suggesting that their gelatinolytic activities are from serine-protease with contemporaneous gelatin-cleavage activity (Figure 4).

Finally, pepstatin A, a reversible aspartic acid protease inhibitor, completely blocked all the higher gelatinases (i.e., 309, 255, 177, 79, and 59 kDa), suggesting that these activities could be due to aspartate proteases (Figure 4).

The substrate specificity of embryo proteases was tested by comparing the proteolytic activities of lysates from both control and V-treated embryos. To this aim, we carried out substrate gel zymography by using casein as a potential substrate. Only one of the nine gelatinases (177 kDa) slightly hydrolyze casein (data not shown), suggesting that they are specific proteases.

## 4. Discussion

In this paper we used the sea urchin *P. lividus* as an embryo model to test whether different V concentrations, ranging from 50 nM (similar to those found in moderately or non-polluted seawater) to 1 mM (very cytotoxic) induce stress during embryo development. Two crucial metal ions related processes were evaluated: the fertilization rate and the activity of proteases.

### 4.1. V Effects on Fertilization Outcomes in a Dose Dependent Manner

A recent paper detailed that metals released by disposed mine tailings, including V, alter fertilization in terms of fertilization membrane quality and the fertilization success of the green sea urchin, *Psammechinus miliaris* [47].

External perturbations due to the action of competing metals such as V, could alter fertilization, making this process a suitable investigative test to evaluate metal toxicity [49].

By treating virgin eggs, a general reduction in the fertilization rate related to the dose of V administered was observed. Most of the eggs treated with high V concentrations fertilized, however a more careful analysis showed anomalies related to egg morphology and/or of the fertilization membrane. Some of the unfertilized eggs also showed evident morphological anomalies.

### 4.2. Modulation of Gelatinase Activity Induced by V in Sea Urchin Embryos

Chiarelli et al. recently demonstrated that V induces dose dependent morphological alterations in 36-h embryos, especially concerning skeleton integrity [16]. The sea urchin embryonic skeleton originates from primary mesenchyme cells, whose migration into the blastocoel, as well as the formation of a syncytial structure in which the mineral matrix is deposited, require the activity of metalloproteases. MMPs are enzymes that have a critical role in tissue remodeling, cell migration, cell differentiation, and morphogenesis during embryonic development [50,51,52], and there is clear evidence that an extracellular matrix environment is crucial for the normal morphogenesis of sea urchin embryos. In fact, MMP inhibitors are known to disrupt the skeleton formation of sea urchin embryos [44].

Gelatin zymography gels have shown that *P. lividus* embryos express nine gelatinases ranging from 309 to 22 kDa, whose levels are influenced by V. In particular, they seem to directly reflect the toxic V effect in a dose dependent manner. The lowest V concentrations (from 50 nM to 1 µM) reduce the gelatinolytic activity of the highest molecular weight proteases (from 309 to 59 kDa), whereas no significant variation compared to the controls were observed for the smallest ones (34–22 kDa). These data are in accordance with previous investigations, which showed an almost normal embryo morphology and only a slight reduction in total skeletal mass [16], confirming that sea urchin embryos are able to tolerate low V concentrations. Increasing the V concentration (from 100 µM to 1 mM) caused more drastic effects as observed by an overexpression of the proteolytic activity mediated by high molecular weight gelatinases (309–59 kDa) and a complete inhibition of low molecular weight ones (34–22 kDa). At a morphological level, these high V doses caused an evident delay in embryo development, abnormal phenotypes, and a drastic reduction in the total skeletal mass [16].

### 4.3. V Induces the Activation of Two MMP-like Gelatinases

Biochemical characterization of the nine identified proteases demonstrated that the 79 and 59 kDa species require both Zn^2+^ and Ca^2+^ to maintain their catalytic activity. Furthermore, their cleavage activity was limited to denatured collagen (gelatin), suggesting that these enzymes may be members of the MMP family. In contrast, the low molecular weight proteases were inhibited by PMSF, suggesting they belong to the serine proteases with coincidental gelatin cleavage activity.

## 5. Conclusions

Our paper demonstrates the V effects on two metal-related crucial processes.

V decreases the fertilization outcome with a marked effect on fertilization membrane morphology.

It is known that during mammalian embryonic development, serine proteases and MMPs are involved in tissue rearrangements and cell migrations [53]. Our previous data suggested that V could perturb embryo morphogenesis and skeleton formation by modifying gelatinases expression. In addition, it was also demonstrated that, after V treatment, the activation of HSPs and autophagy were not sufficient to restore the damage and the embryos activated a process of selective apoptosis, trying to eliminate only the damaged cells [16]. Therefore, it was suggested that this latter process requires a reorganization of the embryonic architecture and this could be mediated by important cellular movements and tissue rearrangements manipulated by the MMPs.

In conclusion, our findings contribute to increasing knowledge on the possible threat of V for the environment and human health. Moreover, this paper, for the first time, highlights new approaches that could detect metal-induced toxicity through biomarkers, reflecting metal ion dependent processes, and shows a clearer picture of the effects of V at the cellular level, with the advantage of studying them not in isolated cells, but in cells normally connected in a model of a whole animal organism.

Since this is the first paper that use a combination of two metal-related aspects, in an embryonic model system, similar studies on other bioindicator models would be suitable to understand if there is a species-specific response or a general response to V exposure.

## Figures and Tables

**Table 1:** Toxicity Index calculated for each V concentration.

V Concentrations	TI
1 mM	0.8
500 µM	0.7
100 µM	0.6
50 µM	0.4
1 µM	0.2
500 nM	0.04
100 nM	0.04
50 nM	0.02

## Data Availability

The data presented in this study are available on request from the corresponding author.
